# Theoretical and experimental investigation of Xenotime-type rare earth phosphate REPO_4_, (RE = Lu, Yb, Er, Y and Sc) for potential environmental barrier coating applications

**DOI:** 10.1038/s41598-020-70648-0

**Published:** 2020-08-13

**Authors:** Jing Han, Yanfei Wang, Rongjun Liu, Fan Wan

**Affiliations:** Science and Technology On Advanced Ceramic Fibers and Composites Laboratory, College of Aerospace Science and Engineering, National University of Defense Technology, Changsha, 410073 P. R. China

**Keywords:** Aerospace engineering, Ceramics, Mechanical properties

## Abstract

The mechanical and thermophysical properties of Xenotime-type REPO_4_ (RE = Lu, Yb, Er, Y and Sc) have been theoretically and experimentally investigated for a potential environmental barrier coating (EBC) topcoat application. The results show that the current studied REPO_4_ exhibits a quasi-ductile property, suggesting a potential long life expectancy of its made coatings. Further, from the study of underlying parameters governing thermophysical properties of a ceramic, low thermal expansion coefficients (TECs) and low thermal conductivities cannot be achieved simultaneously, due to mutual exclusive nature of above two parameters. REPO_4_ has been unveiled to have rather small TECs, attributing partly to its weak lattice anharmonicity, and is thus well-matched with silicon carbide based ceramic matrix composites. Last, the current investigated REPO_4_ exhibits very good high-temperature water vapor corrosion resistance, excellent calcium-magnesium aluminosilicates (CMAS) resistance as well as excellent chemical compatibility with silicon bond coats at elevated temperatures. Therefore, the Xenotime-type rare earth phosphates are a promising EBC topcoat material.

## Introduction

In order to achieve a higher thermal efficiency, according to Carnot Cycle, there is endless driving force to increase the inlet temperature of advanced gas turbines^1–3^. With the increase of operation temperatures, it of course imposes more thermal loads to hot-section components, and hence makes the thermal environment more deteriorate and thus severely challenges corresponding materials. Unfortunately, the conventional nickel based superalloys cannot survive these demanding environments, and silicon carbide based ceramic matrix composites (CMCs) are a promising candidate to replace those superalloys due to a combination of superior properties such as: superior high-temperature mechanical properties, excellent oxidation/thermal shock resistances, high reliability and damage tolerance, low densities, as well as their excellent high temperature stability, which is capable of surviving temperatures higher than 1,400 °C, a temperature well above superalloy’s upper limit^4^. However, one fatal drawback of silicon carbide based CMCs as a gas turbine hot-section component is that they tend to react with high-temperature water vapor, a byproduct of fuel combustion, which results in a rapid recession of CMCs and thus cannot satisfy the reliability and durability criteria for aero-engine application. In this sense, the prevention and protection of silicon carbide based CMCs in high-temperature combustion gases that are both oxidative and rich in water vapor is the core and bottleneck technology^5–7^.

In order to address above problems, there are mainly two strategies. One is to develop a more oxidation and water–vapor resistant CMCs, such as to employ an oxidation and water vapor-resistant compounds to modify both interphase^8,9^ and matrix^10,11^ of CMCs. This work is still under way. The other strategy is more direct and simple, that is to employ a so-called environmental barrier coating, EBC, to physically isolate the harmful combustion gases and CMC components^4–7^. Despite of different functions, EBC is rather similar to thermal barrier coating (TBC), the main function of latter is to provide a thermal insulation so as to increase working temperature of gas turbines. Similar to TBC, EBC has at least two layers, which are bond coat and top coat^5^. The main function of EBC bond coat is to provide sufficient adhesion and oxidation resistance, and silicon is the common choice in the state-of-art^12–14^. The problem of Si bond coat is poor oxidation resistance and limited temperature capability restrained by its melting point, which is around 1,410 °C. To address these issues, a hafnium oxide (HfO_2_) modified Si bond coat is proposed to improve its oxidation resistance by forming a HfSiO_4_ phase^12–14^. Meanwhile, a rare earth silicide compound has been proposed as a high-temperature capable bond coat by a U.S. patent^15^.

On the other hand, as EBC top coats directly contacts with combustion gases, their main function is to improve environmental durability. That is to say, EBC top coats have to be both water vapor and calcium-magnesium aluminosilicates (CMAS) resistant^16,17^. In addition, as EBC is a prime reliant coating, which indicates its failure could perhaps lead to a catastrophic consequence, the reliability and durability are of primary concern^17^. To ensure long durability and good reliability, a low stress level of EBC coating system is mandatory^17^. In this regard, the thermal expansion coefficient (TEC) matching between top coats and substrate CMCs is a top priority. A larger TEC of top coats compared to CMC substrates tend to generate tensile stress, which drives to form mud cracks during thermal cycling^18^. These mud cracks allow hot corrosive combustion gases directly attack bond coats or CMC substrates, leading to their rapid failure^18^. Besides, a good phase stability, low elastic modulus and good sintering resistance of top coats are all favored to produce low stress levels and thus a good lifespan of relevant EBCs^17^.

To date, there are three generations of EBC top coats developed, which are the first generation mullite (i.e. Al_2_O_3_·SiO_2_ oxide mixtures)^2^, the second generation BSAS (i.e. BaO·SrO·Al_2_O_3_·SiO_2_ oxide mixtures)^2,19,20^ and the third generation rare earth disilicates (RE_2_Si_2_O_7_) or monsilicates (RE_2_SiO_5_)^2,19^. If we closely examine these three generations of EBC topcoats, it is easy to find that they are all silicon containing compounds. In addition, despite of different chemical compositions of all these three-generation EBC topcoats, they in fact degrade for the same reason, that is, the volatility of silicon due to water vapor attack, attributing to the weak bonding of Si–O bonds^21^. The implication of this is that, if it is possible to develop a compound without silicon, it perhaps can find a more water vapor resistant EBC top coat. Following this clue, in the current study, we select a rare earth phosphate, REPO_4_, as a potential EBC top coat material. The fact is that P-O bonds in REPO_4_ are much stronger than those of Si–O bonds in the above compounds. Besides, REPO_4_ has already proposed as a potential TBC topcoat as suggested by Feng et al.^22^ and Wang et al.^23^, indicating they possess a good high temperature phase stability, and even more encouragingly, a very good CMAS resistant property^23^. Note that in^22,23^ larger RE cations are employed, forming a monoclinic (or Monazite-type) phase, which has a larger thermal expansion coefficient (from 8–10 × 10^−6^ K^−1^)^22^ and is thus not appropriate for EBC application. By contrast, those RE phosphates with smaller RE cations, such as YPO_4_^24^ and LuPO_4_^21^, tend to form a tetragonal (or Xenotime-type) phase with much smaller TEC values, for instance, 5.9 × 10^−6^ K^−1^ for LuPO_4_^21^. As a result, they are promising EBC topcoats^21,24^.

In the current study, we have systematically investigated the mechanical and thermophysical properties of a Xenotime-type REPO_4_ with smaller RE cations, i.e. RE including Lu, Yb, Er, Y and Sc, for potential EBC topcoat applications. First, we employ first-principle calculations to predict elastic constants of REPO_4_ (RE = Lu, Yb, Er, Y and Sc), on the basis of which the mechanical properties can then be calculated. Second, the thermophysical properties (i.e. thermal expansion coefficients and thermal diffusivities) of REPO_4_ can be measured. From the discussion of underlying parameters dictating those thermophysical properties, it is for the first time unveiled that a low thermal expansion coefficient and a low thermal conductivity are mutually exclusive and thus cannot be achieved simultaneously. Lastly, the water vapor corrosion resistance and chemical compatibility of REPO_4_ with Si bond coat are experimentally studied to justify them as a potential EBC topcoat.

## Methodology

### Theoretical calculation methods

The elastic constants of REPO_4_ (RE = Lu, Yb, Er, Y and Sc) are theoretically predicted based on first principles calculations. The calculations are carried out employing the CASTEP code^25^. The plane wave basis is used under periodic boundary conditions. The kinetic energy cutoff is set to 500 eV for expanding Bloch waves in the reciprocal space. For the energy integrations, a discretized 5 × 5 × 5 k sampling grid is applied in the first irreducible Brillouin zone based on Monkhorst–Pack method^26^. For the exchange correlation energy, polarized generalized gradient approximation (GGA) is used^27^. The crystal structures are fully optimized by independently modifying lattice parameters and internal atomic coordinates. The Broyden–Fletcher–Goldfarb–Shanno (BFGS) minimization scheme29 has been employed to minimize the total energy and interatomic forces. For the pseudo-atoms, the ultra-soft type pseudopotentials are applied for RE, P, and O atoms to account for the electrostatic interactions between valence electrons and ionic cores. The criteria for convergence in geometry optimization are selected as follows: the difference in total energy within 1 × 10^−6^ eV/atom, the ionic Hellmann–Feynman forces within 0.002 eV/Å, the maximum stress within 0.01 GPa and the maximum ionic displacement within 1 × 10^−4^ Å.

### Material preparation and characterization

The thermophysical properties (i.e. thermal diffusivity, thermal expansion coefficient) of REPO_4_ are experimentally measured from the sintered REPO_4_ ceramic bulks. The starting REPO_4_ (RE = Lu, Yb, Er, Y and Sc) powders and Yb_2_SiO_5_, Yb_2_Si_2_O_7_ powders (which are used as a reference to compare water vapor corrosion rates) are all 99.9% pure, purchased from the Kai-Star Electro-Optic Materials, Wuxi, China. The powders were cold isostatic pressed at 50 MPa into disk-shaped (12.7 mm in diameter × 2 mm high) and bar-shaped (5 mm × 5 mm × 25 mm) green compacts. The green compacts were then sintered at 1,500 °C for 20 h in air.

The densities of bulks were measured by Archimedes’ method in distilled water. The phases were examined by X-ray diffraction (XRD, Bruker D8 Advanced, Cu Kα radiation). The thermal diffusivity is measured by means of the laser flash technique, using thermal constant measurement equipment (NETZSCH LFA 427, Bavaria, Germany). The thermal conductivity *k* (in W/m•K) was calculated from the equation:

1 $$k \, = c_{{\text{p}}} D \, \rho$$

where *c*_p_ is the specific heat (in J/kg•K), *D* the thermal diffusivity (in cm^2^/s), and *ρ* the density (in g/cm^3^). The specific heat capacitance is calculated according to the Neumann–Kopp rule^28^ by employing standard *c*_p_ values extracted from^29^. Thermal expansion coefficients (TECs) were obtained from temperature-dependent changes in the length of the specimens from room temperature to 1,350 °C in air as determined using a vertical high-temperature optical dilatometer (ODHT, Modena, Italy).

The water vapor corrosion behaviors of sintered ceramic bulks were investigated in 50% H_2_O/50% O_2_ water vapor flowing at a rate of 0.30 cm/s with an atmospheric pressure at 1,500 °C for 80 h. The water vapor was introduced to an alumina tube by O_2_ carrier gas bubbling through distilled water heated at 81.7°C^30^. For each compound, at least 3 samples were measured. The chemical compatibility of REPO_4_ with conventional silicon bond coat was evaluated by identifying phase compositions of REPO_4_ and Si powder mixtures with a weight ratio 7:3 after dwelling at 1,350 °C in air for 20 h.

## Results

### Crystal structure, phase compositions and densities of REPO_4_ (RE = Lu, Yb, Er, Y and Sc)

**Figure 1:**
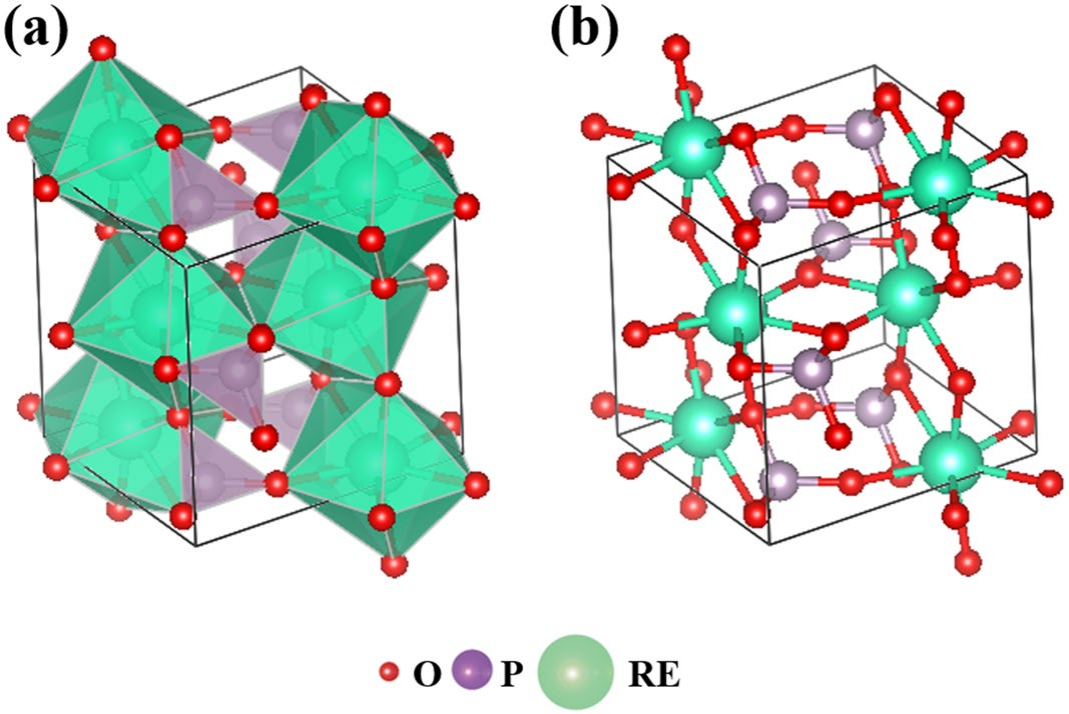
Crystal structure of rare earth phosphate REPO_4_.

Figure 1 shows the typical crystal structure of Xenotime-type rare earth phosphate REPO_4_. As shown, REPO_4_ exhibits a tetragonal structure consisting of two types of polyhedra, i.e. PO_4_ tetrahedra and REO_8_ dodecahedra. In addition, the REPO_4_ crystals can be considered as the accumulation of vertex-connected PO_4_ tetrahedra and REO_8_ dodecahedra. In PO_4_ tetrahedra, the P atom is surrounded by four O atoms; whereas, in REO_8_ polyhedra, the RE atom is surrounded by eight O atoms. As shown in Fig. 1b, each oxygen atom connects two RE atoms and one P atom; whereas, each RE atom or P atom solely connects oxygen atoms, with the former connected to eight oxygen atoms and latter four respectively.

**Figure 2:**
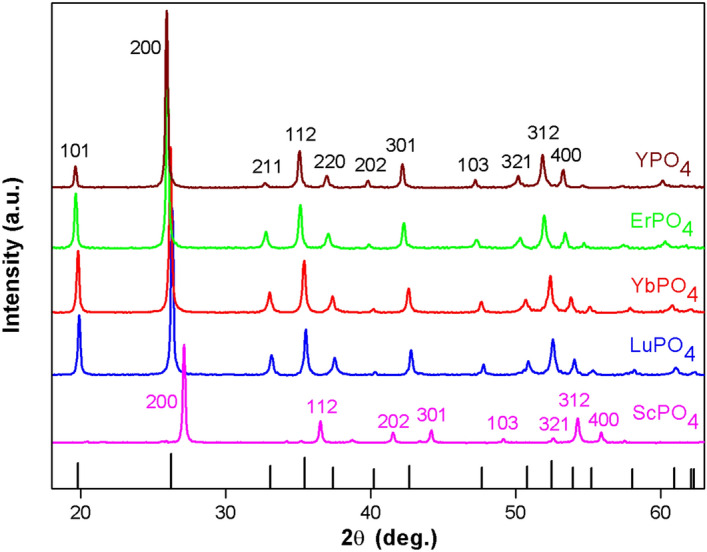
XRD patterns of sintered REPO_4_ ceramic bulks (RE = Lu, Yb, Er, Y and Sc). ScPO_4_ shows a pattern dramatically shifted to higher 2θ angles, attributing to a much smaller cationic size of Sc^4+^ than the other four rare earth elements.

Figure 2 shows the XRD patterns of sintered REPO_4_ (RE = Lu, Yb, Er, Y and Sc) ceramic bulks. The measured patterns of REPO_4_ are compared with the standard XRD spectra of LuPO_4_ (ICDD PDF No. 43-0003), suggesting that single REPO_4_ phases have been formed. With an increase of ionic radius of rare earth element (from Sc to Y), the diffraction peaks are expected, according to the Bragger’s law, to shift to lower angle. It is worth pointing out that, whereas the cationic sizes of Lu^3+^, Yb^3+^, Er^3+^, Y^3+^ are more or less in the same order, the ionic size of Sc^3+^ is considerably smaller than that of the above four cations. As a result, ScPO_4_ shows an XRD pattern dramatically shifted to higher 2θ angles, which is distinctive from the XRD patterns of the other four rare earth phosphates. Table 1 shows the measured, theoretical and relative densities of REPO_4_ (RE = Lu, Yb, Er, Y and Sc). As shown, the sintered pellets have achieved a high relative density, more than 97% of theoretical density.

**Table 1:** The measured, theoretical and relative density of REPO_4_ (RE = Lu, Yb, Er, Y and Sc).

Compound	ScPO_4_	YPO_4_	ErPO_4_	YbPO_4_	LuPO_4_
Experimental density (g/cm^3^)	3.78	4.38	6.05	6.95	6.02
Theoretical density (g/cm^3^)	3.89	4.48	6.20	7.10	6.17
Relative density (%)	97.2	97.7	97.6	97.9	97.5

### The predicted elastic constants of REPO_4_ (RE = Lu, Yb, Er, Y and Sc) from first principles calculations

Table 2 shows the predicted elastic constants of REPO_4_ (RE = Lu, Yb, Er, Y and Sc) from first-principle calculations. As shown, no negative *C*_ij_ value is obtained for these compounds, suggesting that these crystal structures are all stable. For those tetragonal structures such as the current Xenotime-type rare earth phosphates, *C*_22_ = *C*_11_ and *C*_55_ = *C*_11_. As shown in Table 2, the values of *C*_11_ and *C*_22_ are lower than those of *C*_33_, indicating that the chemical bonds are identical in the directions of [100] and [010] but they are weaker than those in the [001] direction for current studied REPO_4_ compounds. In fact, both RE–O and P–O bonds in Xenotime-type REPO_4_ crystal structures can be divided into two groups, which are longer and shorter bonds respectively. Understandably, those shorter RE–O and P–O bonds tend to generate a stronger covalent character than those longer ones^31^. Referring to the crystal structure of REPO_4_, the shorter bonds are aligned mainly along the [001] direction. As a result, the calculated *C*_33_ values are larger than those of *C*_11_ and *C*_22_.

**Table 2:** Elastic constants *C*_ij_ (in GPa) of REPO_4_ (RE = Lu, Yb, Er, Y and Sc) from first principles calculations.

Compound	ScPO_4_	YPO_4_	ErPO_4_	YbPO_4_	LuPO_4_
*C*_11_	285.9	399.2	265.7	245.7	326.9
*C*_12_	22.1	18.5	28.8	26.1	32.6
*C*_13_	89.5	80.2	96.3	81.0	111.0
*C*_22_	285.9	399.2	265.7	245.7	326.9
*C*_33_	300.0	407.7	367.1	326.5	403.8
*C*_44_	93.6	71.3	78.5	68.3	80.4
*C*_55_	285.9	399.2	265.7	245.7	326.9
*C*_66_	41.9	32.8	22.5	19.8	31.9

For the pure shear *C*_ij_ constants, the present calculations show that, for all REPO_4_ structures currently investigated, *C*_55_ and *C*_44_ are significantly larger than *C*_66_, suggesting that there are probably soft P-O and RE–O bonds on the (001) plane but rigid P-O and RE–O bonds on the (001) and (010) planes^32^. It further indicates shear deformation is easier to take place on the (001) plane. For the other non-diagonal elastic constants, their values are relatively small. The off-diagonal elements also reflect the deviation of atomic force constants from those of central type^33^. For the crystal dominated by central forces, Cauchy’s relation implies that *C*_12_ = *C*_66_, *C*_13_ = *C*_55_, *C*_23_ = *C*_44_ and *C*_25_ = *C*_46_. Applying these conditions to REPO_4_ compounds, they exhibit weak many-body forces such as angular and torsional interactions. To sum up above discussions, for Xenotime-type REPO_4_ compounds, tensile and compressive deformation takes place primarily along the [100] and [010] directions whilst shear deformation takes place primarily on the (001) plane.

### The measured thermophysical properties of REPO_4_ (RE = Lu, Yb, Er, Y and Sc)

#### Thermal diffusivity/ conductivity of REPO_4_ (RE = Lu, Yb, Er, Y and Sc)

**Figure 3:**
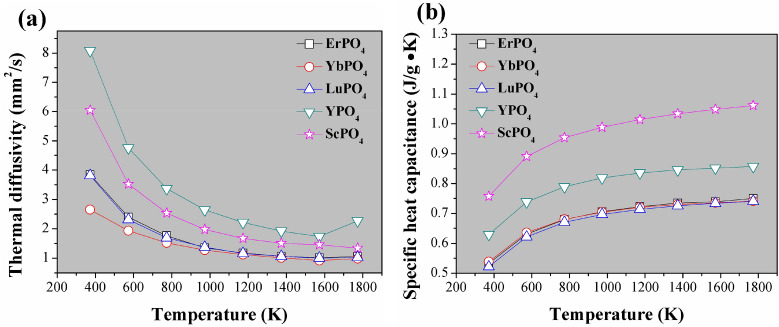
The measured thermal diffusivity (**a**) and calculated specific heat capacitance (**b**) of REPO_4_ (RE = Lu, Yb, Er, Y and Sc) according to Neumann–Kopp rule.

**Figure 4:**
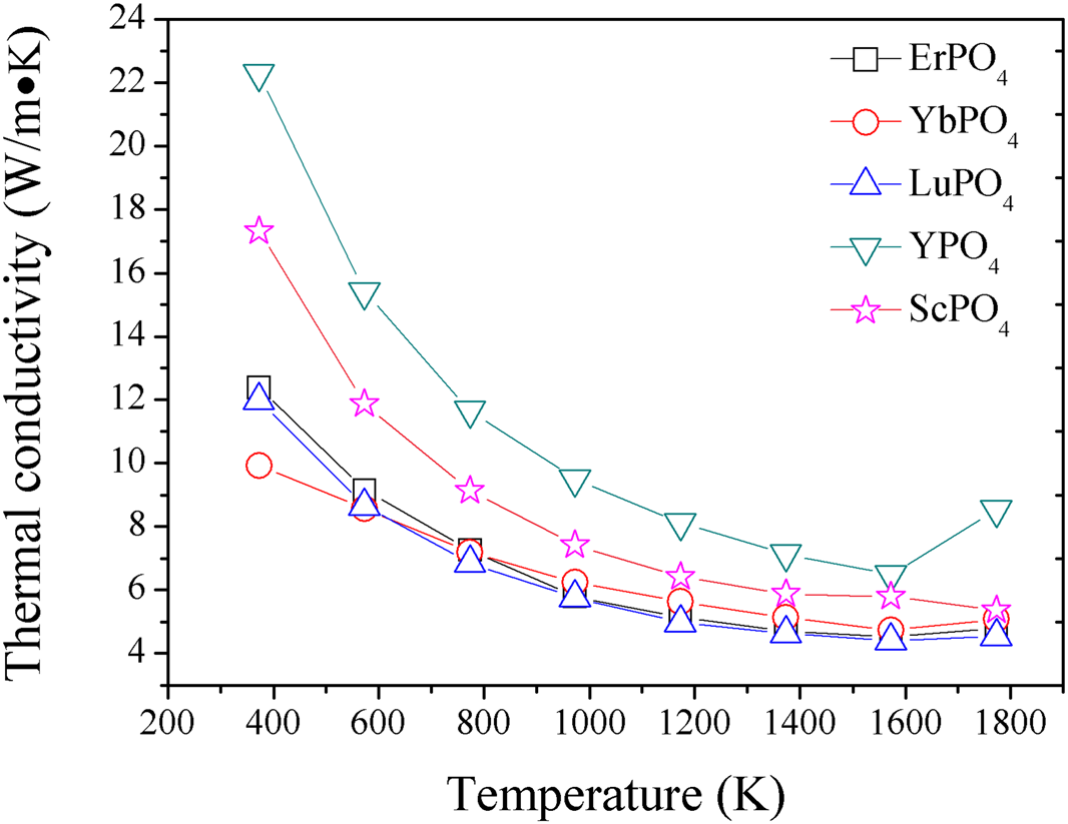
The measured thermal conductivity of REPO_4_ (RE = Lu, Yb, Er, Y and Sc).

Figure 3 shows the thermal diffusivity (a) and calculated specific heat capacitance (b) of REPO_4_. As shown, rare earth phosphates REPO_4_ exhibit thermal diffusivities lower than 3 mm^2^/s from 1,200 K to 1773 K. By using density values (Table 1) and thermal diffusivity and specific heat capacitance shown in Fig. 3b, thermal conductivities of REPO_4_ can be calculated according to Eq. (1), and are shown in Fig. 4. From it, the following trends can be found. First, the thermal conductivities of REPO_4_ are generally decreasing with an increase of temperature. This is a typical feature for a ceramic whose heat transport is dictated by the phonon–phonon Umklapp scattering. Second, a heavier rare earth element of REPO_4_, for instance, RE = Er, Yb, Lu, tends to generate lower thermal conductivity than those of lighter rare earth elements. Indeed, these three REPO_4_ compounds with heavier rare earth elements have almost overlapping thermal conductivity- temperature curves, i.e. very similar thermal conductivity values.

#### Thermal expansion coefficient of REPO_4_ (RE = Lu, Yb, Er, Y and Sc)

**Figure 5:**
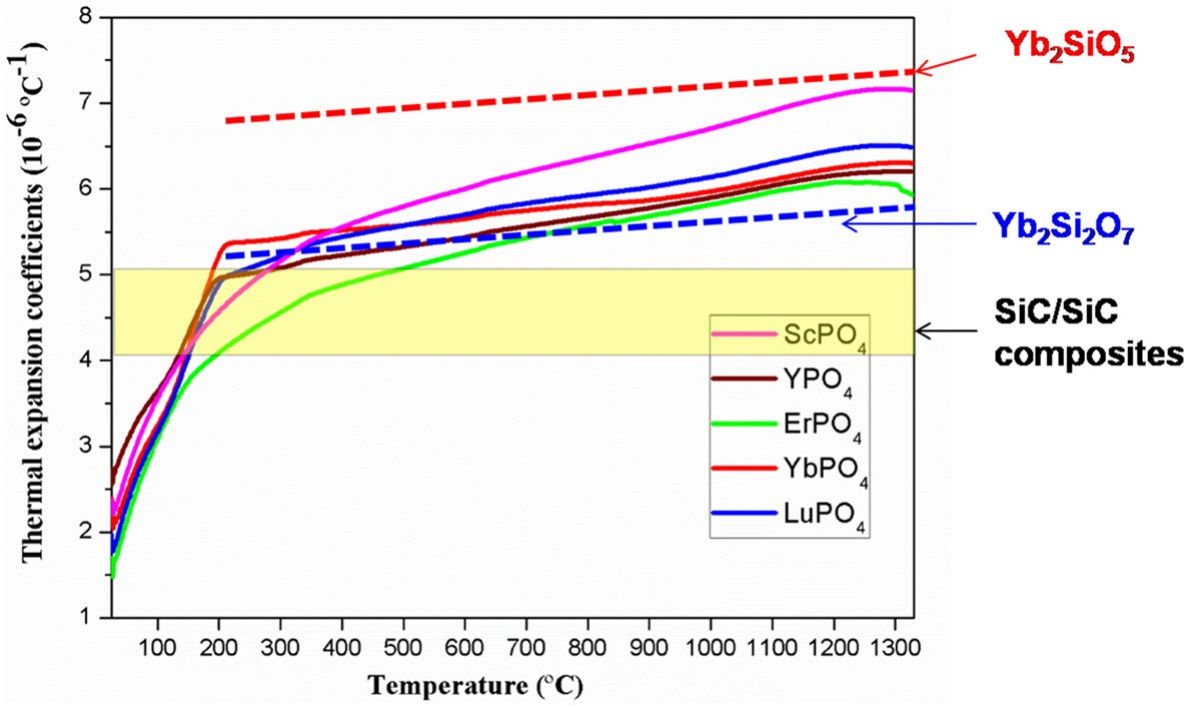
The measured thermal expansion coefficients of REPO_4_ (RE = Lu, Yb, Er, Y and Sc). As a comparison, TEC of typical SiC-based CMCs (the horizontal bands)^4^, TECs of Yb_2_Si_2_O_7_ and Yb_2_SiO_5_^34^ (the dashed lines) are also included.

Figure 5 exhibits the measured thermal expansion coefficients of REPO_4_ (RE = Lu, Yb, Er, Y and Sc) ceramic bulks. As a comparison, the TECs of typical SiC-based CMCs^4^, Yb_2_Si_2_O_7_ and Yb_2_SiO_5_^34^, representatives of third generation EBC topcoat materials, are also included. As shown, the TECs of REPO_4_ are rather close to those of Yb_2_Si_2_O_7_, a compound that is thought to have good TEC matching with SiC based CMCs, suggesting that REPO_4_ currently investigated probably has a good TEC matching with SiC based CMCs.

## Discussions

From the perspective of potential EBC applications, the following three aspects have to be taken into accounts. Firstly, from the mechanical aspect, a smaller elastic modulus together with a quasi-ductile behavior of an EBC topcoat material tends to produce a longer lifespan of its made coatings. From the elastic constants as unveiled in “The predicted elastic constants of REPO_4_ (RE = Lu, Yb, Er, Y and Sc) from first principles calculations” section, different kinds of modulus (or mechanical parameters) can be calculated, such as bulk (*B*), shear (*G*), elastic (*E*) modulus respectively, as well as Poisson’s ratio, *B*/*G* ratio, the latter two of which usually hints the extent of ductility of a material. Secondly, the thermophysical properties of EBC topcoat are usually of prior concern. The preliminary results in “The measured thermophysical properties of REPO_4_ (RE = Lu, Yb, Er, Y and Sc)” section suggests a heavy rare earth element of REPO_4_ tends to generate lower thermal conductivity, whilst a smaller rare earth element of REPO_4_ (i.e. ScPO_4_) tends to have a higher TEC. The underlying mechanisms need further investigation. Last but not least, the water vapor resistance, the thermochemical compatibility issues of REPO_4_ have to be examined and justified as a potential EBC topcoat candidate. The following parts are to be discussed from the above three aspects.

### Mechanical properties of REPO_4_ (RE = Lu, Yb, Er, Y and Sc)

As shown in Table 2, the elastic constants *C*_ij_ can be obtained from first principle calculations. In fact, REPO_4_ has 13 independent elastic constants, i.e., *C*_11_, *C*_22_, *C*_33_, *C*_44_, *C*_55_, *C*_66_, *C*_12_, *C*_13_, *C*_23_, *C*_15_, *C*_25_, *C*_35_ and *C*_46_. Based on these elastic constants, the bulk modulus *B*, shear modulus *G* and Young’s modulus *E* of REPO_4_ can be further calculated. According to Voigt approximations^35–37^, the bulk and shear moduli can be calculated from elastic constants as:

2 $${\text{B}}_{{\text{V}}} = \frac{1}{9}\left( {{\text{C}}_{11} + {\text{C}}_{22} + {\text{C}}_{33} } \right) + \frac{2}{9}({\text{C}}_{12} + {\text{C}}_{13} + {\text{C}}_{23} ,$$

and

3 $${\text{G}}_{{\text{V}}} = \frac{1}{15}\left( {{\text{C}}_{11} + C_{22} + C_{33} - C_{12} - C_{13} - C_{23} } \right) + \frac{2}{5}(C_{44} + C_{55} + C_{66} ;$$

By contrast, on the basis of Reuss approximation, the bulk and shear moduli can be calculated from compliance matrix components as:

4 $${\text{B}}_{{\text{R}}} = \frac{1}{{\left( {{\text{s}}_{11} + {\text{s}}_{22} + {\text{s}}_{33} } \right) + 2({\text{s}}_{12} + {\text{s}}_{13} + {\text{s}}_{23} }},$$

and

5 $${\text{G}}_{{\text{R}}} = \frac{1}{{4\left( {s_{11} + s_{22} + s_{33} - s_{12} - s_{13} - s_{23} } \right) + 3(s_{44} + s_{55} + s_{66} }} ,$$

where *S*_ij_ refers to the components of the elastic compliances that can be obtained through the inversion of the elastic constants (*S*_ij_ = *C*_ij_^−1^) tensor. Both the Voigt and Reuss averaging methods assume that strains and stresses are continuous in polycrystals and can produce respectively the upper and lower bounds of the effective bulk and shear moduli for polycrystals. On the contrary, the Voigt–Reuss–Hill (VRH) approach combines the upper and lower bounds, assuming that the average Voigt and Reuss elastic moduli are a good approximation of the macroscopic elastic moduli. The bulk modulus *B*_VRH_ and shear modulus *G*_VRH_ based on Voigt–Reuss–Hill approximation can be calculated as follows:

6 $${\text{B}}_{{{\text{VRH}}}} = \frac{1}{2} \left( {{\text{B}}_{{\text{V}}} + {\text{B}}_{{\text{R}}} } \right),$$

7 $${\text{G}}_{{{\text{VRH}}}} = \frac{1}{2} \left( {{\text{G}}_{{\text{V}}} + {\text{G}}_{{\text{R}}} } \right).$$

In the following context, we employ *B*_VRH_ and *G*_VRH_ as the calculated bulk modulus and shear modulus. In addition, according to Ref.^38^, the Young’s modulus *E* and Poisson’s ratio ν can be calculated on the basis of *B*_VRH_ and *G*_VRH_^39^ as follows:

8 $${\text{E}} = \frac{{9{\text{B}}_{{{\text{VRH}}}} {\text{G}}_{{{\text{VRH}}}} }}{{3{\text{B}}_{{{\text{VRH}}}} + {\text{G}}_{{{\text{VRH}}}} }},$$

9 $$\upnu = \frac{{3{\text{B}}_{{{\text{VRH}}}} - 2{\text{G}}_{{{\text{VRH}}}} }}{{2\left( {3{\text{B}}_{{{\text{VRH}}}} + {\text{G}}_{{{\text{VRH}}}} } \right)}}.$$

**Figure 6:**
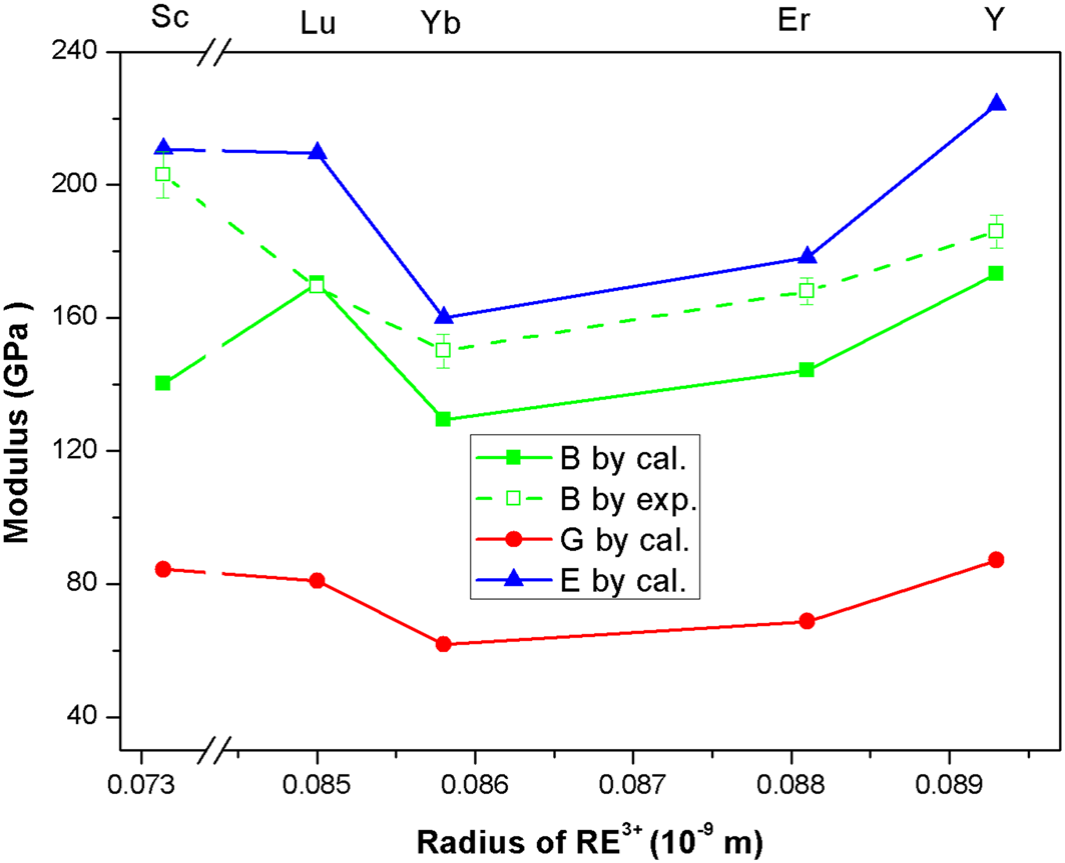
The plot of calculated bulk (*B*), shear (*G*) and Young’s (*E*) moduli of REPO_4_ versus cationic radius of RE^3+^.

Table 3 shows the calculated bulk modulus *B*, shear modulus *G*, and Young’s modulus *E*, Poisson’s ratio *v*, and *B*/*G* ratio. As a comparison, the measured bulk moduli of rare earth phosphates are also included. Figure 6 plots the variation of bulk, shear and Young’s modulus of REPO_4_ versus the radius of rare earth elements. From it, the following trends can be found. Firstly, with an increase of ionic radius of rare earth element RE^3+^, except for the calculated bulk modulus of ScPO_4_, the calculated Young’s, bulk and shear modulus decreases till Yb, and then increases. In other words, YbPO_4_ is predicted to have the lowest above three moduli. Interestingly, this trend is perfectly conforming to the measured bulk modulus from other work^32,38,40,41^ as shown the dash curve in Fig. 6, which confirms the validity of current calculation. The only discrepancy lies in that a much lower bulk modulus of ScPO_4_ is predicted compared to the measured value.

**Table 3:** Elastic moduli *B*, *G*, and *E* (in GPa), Poisson’s ratio *ν*, B/G ratio, and Vickers hardness *H*_V_ (in GPa) in the Hill approximation. Experimental bulk modii are included as a comparison.

Compound	ScPO_4_	YPO_4_	ErPO_4_	YbPO_4_	LuPO_4_
*B*	140.2	173.2	144.1	129.3	170.4
*B*^exp^	203 ± 7^40^	186 ± 5^40^	168 ± 4^32^	150 ± 5^41^	169.3^38^
*G*	84.4	87.2	68.8	61.8	80.9
*E*	210.8	224.1	178.1	160.0	209.6
*ν*	0.25	0.28	0.29	0.29	0.29
*B/G*	1.66	1.99	2.09	2.09	2.11

In addition, if we closely examine the Poisson’s ratio and *B*/*G* ratio, we can find rare earth phosphates REPO_4_ are rather ductile and even show some plastic deformation that is rare for ceramics, which is desirable for EBC application. As we know, the Poisson's ratio reflects the capability of a material to resist deformation in different directions, and a greater Poisson's ratio usually yields a better plasticity and ductility of a material^42^. For instance, the Poisson's ratio of metals is usually greater than 0.3. By contrast, the Poisson’s ratio of ceramics is usually below 0.3. For example, the Poisson's ratios of zirconia (ZrO_2_), lanthanum zirconate (La_2_Zr_2_O_7_) and alumina are 0.27, 0.25 and 0.23 respectively. Except for ScPO_4_, the Possion’s ratios of the other rare earth phosphate currently investigated are in the range of 0.28–0.29, which is comparable to most intermetallic compounds. This suggests REPO_4_ are relatively plastic and ductile.

Furthermore, the B/G value further supports the above conclusion. The B/G value can be taken as a criterion to distinguish a material whether it has a good ductility or not. In general, materials with a B/G value below 1.75 tend to have poor ductility, whereas, materials with a B/G value above 2.0 tend to have excellent ductility. The B/G values of REPO_4_ except ScPO_4_ are in a range of 1.99–2.11, which lies in the plastic and ductile region. The Poisson’s ratio and B/G value of ScPO_4_ are 0.25 and 1.66 respectively, indicating that it might possess a typical feature of ceramic brittleness. As a comparison, the Poisson’s ratio and B/G value of RE_2_SiO_5_ are in the range of 0.20–0.25 and 1.46–1.73 respectively^43^, which are lower than values of REPO_4_, suggesting they exhibit a more brittle nature than currently investigated REPO_4_. By contrast, the Poisson's ratio and B/G value of RE_2_Si_2_O_7_ are in the range of 0.30–0.31 and 2.33–2.35^44^ respectively, suggesting that RE_2_Si_2_O_7_ might possess even better behavior than REPO_4_ in terms of toughness. In fact, it is worth pointing out that, due to such quasi-ductile behavior of RE_2_Si_2_O_7_ and REPO_4_, they have been proposed as a novel interphase candidate for SiC/SiC interphase so as to improve oxidation resistance of interphase^8,9^, replacing conventional layered PyC or BN which are highly susceptible to oxidation at low temperatures.

To sum up, in terms of modulus, YbPO_4_ exhibits lowest value, which perhaps suggests it might provide best strain tolerance of its made coating and is thus desirable for EBC topcoat application. Meanwhile, from quasi-ductile perspective, ErPO_4_, YbPO_4_ and LuPO_4_ exhibit excellent behavior, suggesting they might produce durable coatings.

### Thermophysical properties of REPO_4_ (RE = Lu, Yb, Er, Y and Sc)

**Figure 7:**
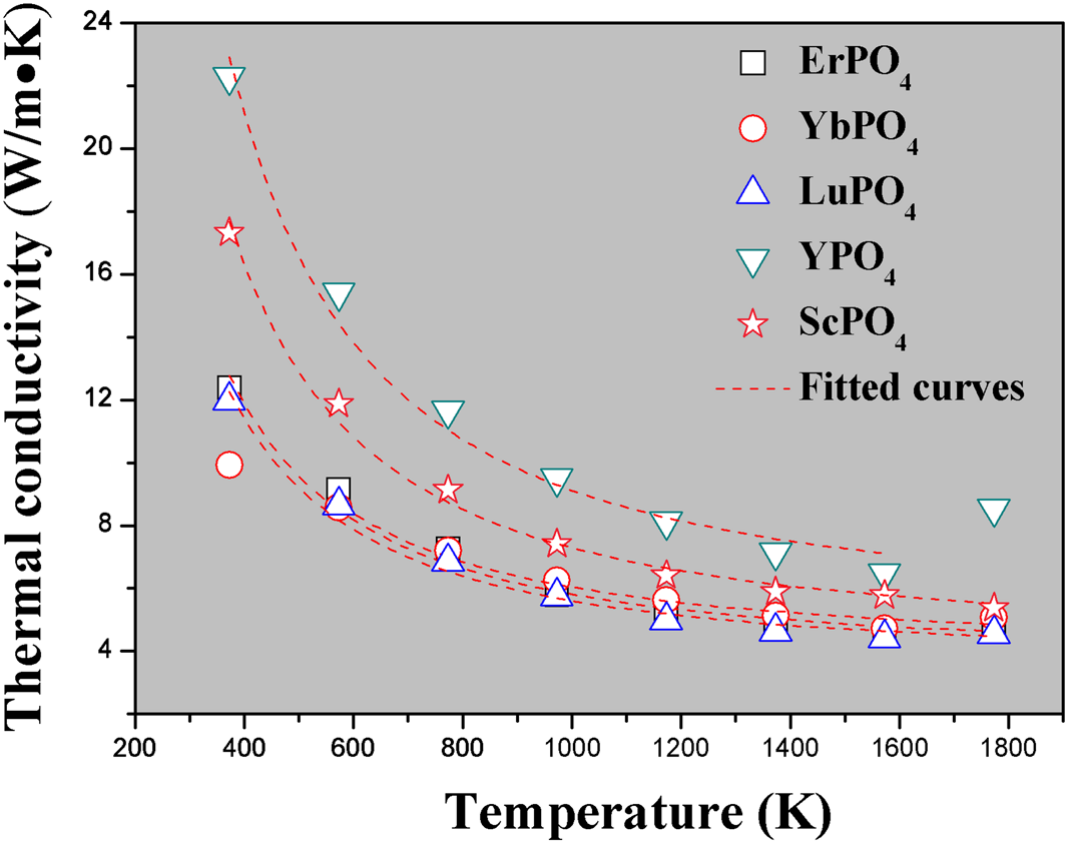
The fitted curves of thermal conductivity versus temperature according to Eq. (10) for REPO_4_ (RE = Er, Yb, Lu, Y, Sc).

According to^45–47^, the Grüneisen parameter γ, which is a reflection of lattice anharmonicity, is closely related to the thermophysical properties of a material, such as thermal conductivity and thermal expansion coefficient. Based on the formalism developed in^45^, the Grüneisen parameter γ can be calculated according to the following equation:

10 $$\upgamma = Ma\omega_{D}^{3} /(2664.8 \times A)$$

in which *M*, *a*, *ω*_*D*_ are average atomic mass, size in the lattice and Debye characteristic frequency respectively, while A is a parameter that can be obtained by curve fitting of thermal conductivity (*k*) versus temperature (T) curves (refer to Fig. 7) according to Eq. (11):

11 $$k = \frac{A}{{3{\text{T}}_{1} }} + \frac{{2A\sqrt {{\text{T}}_{1} } }}{{3{\text{T}}^{1.5} }},$$

where A and T_1_ are characteristic parameters. In fact, Eq. (11) depicts the thermal conductivity of a lattice without any point defects, where phonon–phonon Umklapp scattering is the dictating factor to define its thermal conductivity. In fact, according to^45,46^, A/3T_1_ in Eq. (11) represents the minimal lattice thermal conductivity, *k*_min_, neglecting thermal radiation effects, and can be further expressed as follows:

12 $$k_{{\min }} = \frac{{\text{A}}}{{3{\text{T}}_1}} = \xi \sqrt {\frac{{\text{E}}}{{{\text{aM}}\gamma }}} ,$$

where *ξ* is a constant. From Eq. (12), it is apparent that, a material with higher average atomic mass (i.e. bigger *M*) and stronger lattice anharmonicity (i.e. larger γ values) tends to generate lower thermal conductivity. This perhaps explains ErPO_4_, YbPO_4_ and LuPO_4_ have lower thermal conductivities than YPO_4_, ScPO_4_, as Er, Yb and Lu have much heavier atomic mass than Y and Sc.

Table 4 shows some basic parameters of REPO_4_ and the related calculation method of each parameter has been attached under the table. As shown, from the fitting of k-T curves (which yields A and T_1_) and the calculation of other related physic parameters, the Grüneisen parameters can be obtained according to Eq. (10). It is found that, the currently studied rare earth phosphates all exhibit very small Grüneisen parameters.

**Table 4:** Some basic parameters of REPO_4_ (RE = Lu, Yb, Er, Y, Sc).

	*M*^a^ (10^−26^ kg)	*a*^3b^ (10^−29^ m^3^)	*a*^b^ (Å)	ρ^b^ (× 10^3^ kg/m^3^)	*v*^c^ (m/s)	*ω*_D_ (× 10^13^ Hz)^d^	*θ*_D_ (K)^e^	A (W/m)^f^	T_1_ (K)^f^	γ^g^	*k*_min_^h^
LuPO_4_	7.471	1.211	2.296	6.17	4,643	7.882	601.9	4,563.4 ± 101.5	420.2 ± 28.1	0.69	3.62
YbPO_4_	7.417	1.044	2.186	7.10	3,883	6.924	528.7	4,882.1 ± 150.9	418.3 ± 29.8	0.41	3.89
ErPO_4_	7.256	1.170	2.270	6.20	3,724	6.394	488.3	4,753.8 ± 136.5	427.7 ± 37.8	0.34	3.70
YPO_4_	5.089	1.136	2.248	4.48	5,785	10.031	766.0	8,264.7 ± 173.6	545.2 ± 45.8	0.52	5.05
ScPO_4_	3.873	0.996	2.151	3.89	6,025	10.918	833.7	6,444.2 ± 92.7	507.5 ± 24.5	0.63	4.23

^a^Average atomic mass in REPO_4_ lattice;

^b^Average atomic volume in the REPO_4_ lattice, can be calculated as: *a*^3^ = *M*/*ρ*, in which *ρ* is theoretical density of each compound; *a* is average atomic size;

^c^Sound speed. $v = \frac{1}{3}(\frac{2}{{{\text{v}}_{{\text{l}}}^{3} }} + \frac{1}{{{\text{v}}_{{\text{t}}}^{3} }})^{ - 1/3}$, while $v_{l} = \sqrt {\left( {{\text{B}} + \frac{4}{3}{\text{G}}} \right)/{\uprho }}$; $v_{t} = \sqrt {{\text{G}}/{\uprho }}$^22^, where *B* and *G* are bulk modulus and shear modulus respectively, and are used values in Table 3;

^d^Debye frequency, is calculated according to *ω*_*D*_ = (6π^2^)^1/3^*v*/*a*^45^;

^e^Debye temperature, is calculated according to *θ*_*D*_ = *ħω*_*D*_/*k*_*B*_; in which *ħ* and *k*_*B*_ are Planck constant and Boltzmann constant respectively;

^f^Are fitted values from curve fitting in Fig. 7;

^g^Grüneisen parameters, representing lattice anharmonicity, is calculated according to: γ = *Maω*_*D*_^3^/(2,664.8 × A);

^h^*k*_min_ = A/3T_1_.

**Figure 8:**
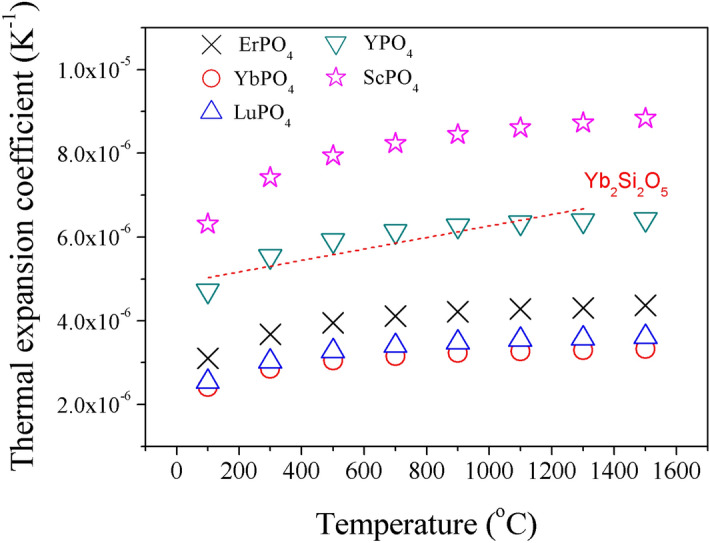
The calculated TEC of REPO_4_ according to Eq. (12) on the basis of measured specific heat capacitance (refer to Fig. 3b). As a reference, the measured TECs of Yb_2_Si_2_O_7_^34^ have also been included.

In fact, the thermal expansion coefficient *α* of a material has the following relationship with Grüneisen parameter^48^:

13 $$\alpha = c_{{\text{P}}} \rho \gamma /3B$$

where *c*_P_ is specific heat capacitance (in J/g K), *ρ* is density (in g/cm^3^), *B* is the bulk modulus (in GPa) and γ is the Grüneisen parameter. On the basis of Eq. (13), by employing the measured *c*_P_ values (Fig. 3b), we can calculate thermal expansion coefficients of REPO_4_ (Fig. 8), by using *ρ*, *γ* values from Table 4 and *B* values from Table 3.

As shown in Fig. 8, the calculated TECs are rather close to those measured ones (refer to Fig. 5) which are in principle lying between curves of Yb_2_SiO_5_ and Yb_2_Si_2_O_7_, suggesting the validity of the current methodology. It is worth pointing out that both measured and calculated TEC values indicate ScPO_4_ has highest value whilst those rare earth phosphates REPO_4_ with heavier atomic mass have lower values. The discrepancy of these calculated and measured TEC values might originate from the slight temperature dependence of density *ρ* and bulk modulus *B*, which is neglected in current calculations.

By comparing Eqs. (12) and (13), it is again affirmed that the Grüneisen parameter *γ* has an important role in determining both thermal conductivity *k* and thermal expansion coefficient *α*. In addition, the modulus, which is a reflection of bond strength, is another parameter affecting both *k* and *α*. Different from thermal barrier coating application, which requires a topcoat material desirably having lower *k* and higher *α*, EBC topcoat material needs to have lower *k* but lower *α*, as a result of relatively low TEC of common SiC based CMCs (refer to Fig. 5). According to Eqs. (12) and (13), theoretically a low thermal conductivity and high thermal expansion (which is desirable for TBC application) can be achieved simultaneously. However, unfortunately, a low thermal conductivity and low thermal expansion coefficient are exclusive, and thus cannot be achieved simultaneously. This suggests that, for the selection of EBC topcoat material, a compromise of *k* and *α* (or, a compromise of lattice anharmonicity and bond strength) would be recommended, which is distinctive from the selection of TBC topcoat material where materials with strong lattice anharmonicity and weak bonding are favored.

### The justification of REPO_4_ (RE = Lu, Yb, Er, Y and Sc) for potential EBC topcoat application

Except the mechanical and thermophysical properties, the high temperature stability, the CMAS resistance, as well as water vapor resistance of a candidate EBC topcoat are also important factors. Hence, we discuss these properties of xenotime-type REPO_4_ (RE = Lu, Yb, Er, Y and Sc) as follows.

**Figure 9:**
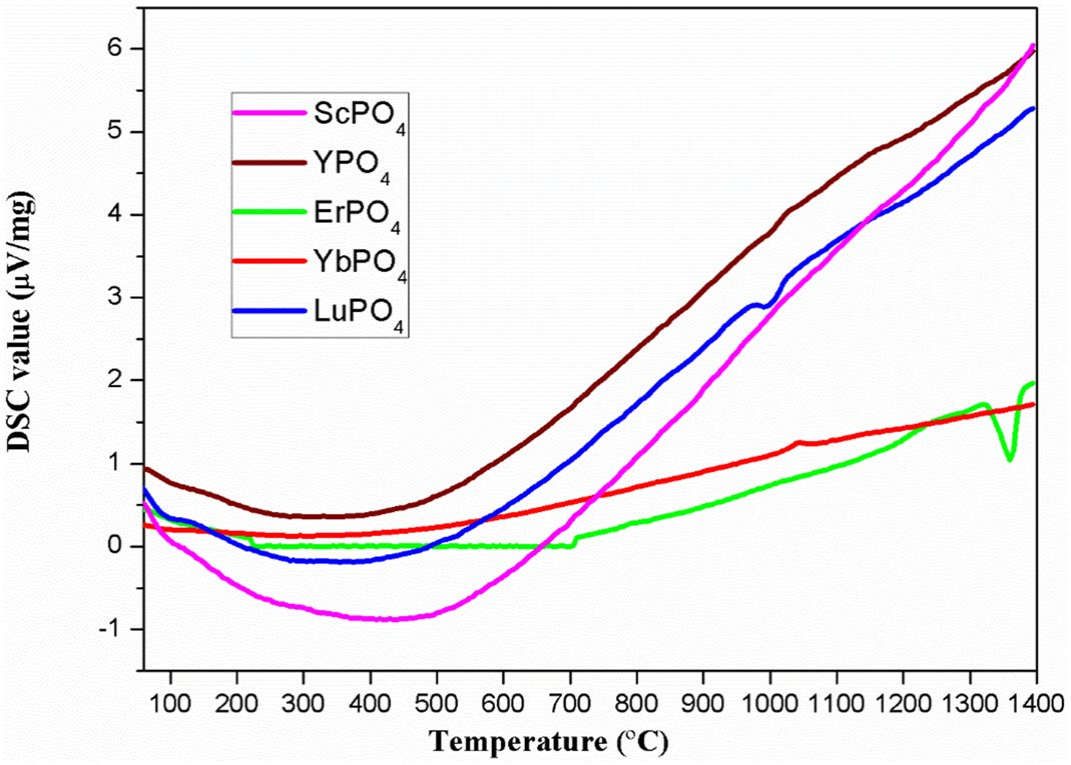
The DSC curves of REPO4 powders (RE = Lu, Yb, Er, Y and Sc).

As discussed in^21–23^, rare earth phosphates exhibit excellent CMAS resistance, attributed to a dense and crack-free layer formed on the surface of REPO_4_ as a result of their reaction with molten CMAS. These dense layers suppress the further penetration of CMAS melts^[^^23^. In addition, the xenotime-type REPO_4_ (RE = Lu, Yb, Er, Y and Sc) exhibits very good high-temperature stability, as shown in Fig. 9, which illustrates the DSC curves of current investigated rare earth phosphates measured in ambient atmosphere up to 1,400 °C. As shown, there are no endothermic or exothermic peaks detected for REPO_4_ (RE = Lu, Yb, Y and Sc), suggesting they have very good phase stability up to 1,400 °C. However, for ErPO_4_, there is a peak around 1,350 °C, suggesting that there might be a phase transformation around this temperature.

As a potential EBC topcoat material, the water vapor corrosion resistance is a key factor. Table 5 exhibits the water vapor corrosion rates of REPO_4_ at a normalized condition of 50% H_2_O-balance O_2_ vapor with a flow of 0.3 cm/s and a total pressure of 1 atm at 1,500 °C for 80 h. For comparison, the values of Yb_2_SiO_5_ and Yb_2_Si_2_O_7_ are also included. As shown, the current investigated REPO_4_ has better water vapor corrosion resistance compared to third generation rare earth silicates.

**Table 5:** Comparison of corrosion rate constants of REPO_4_ (RE = Lu, Yb, Er, Y and Sc). As a comparison, the values of Yb_2_SiO_5_ and Yb_2_Si_2_O_7_ are also included. The normalized conditions are 50% H_2_O-balance O_2_ vapor with a flow of 0.3 cm/s and a total pressure of 1 atm at 1,500 °C for 80 h.

Compound	ScPO_4_	YPO_4_	ErPO_4_	YbPO_4_	LuPO_4_	Yb_2_SiO_5_	Yb_2_Si_2_O_7_
Corrosion rate constants (× 10^−4^ mg/ cm^2^ h)	2.32 ± 0.91	2.51 ± 1.33	4.08 ± 1.87	4.31 ± 1.92	1.89 ± 1.03	7.46 ± 3.24	11.02 ± 3.91

Regarding the data shown in Table 5, there is a concern raised from the execution of high temperature water vapor test by use of an alumina tube furnace. Unfortunately, a reaction product Al_5_RE_3_O_12_ has been found on the surface of bulk samples after high temperature water vapor corrosion test. The formation mechanism of byproduct Al_5_RE_3_O_12_ is likely to be two steps as shown in the Chemical Formula (R1) and (R2). Firstly, the solid Al_2_O_3_ from alumina tube reacts with water vapor to form gaseous Al(OH)_3_. Secondly, the gaseous Al(OH)_3_ then reacts with REPO_4_ to form solid Al_5_RE_3_O_12_**.** By combining Chemical Formula (R1) and (R2), we then can reach Chemical Formula (R3).

R1 $$2.5{\text{ Al}}_{2} {\text{O}}_{3} \,\left( {\text{s}} \right) + 7.5{\text{H}}_{2} {\text{O}}\,\left( {\text{g}} \right) \to 5{\text{Al}}\left( {{\text{OH}}} \right)_{3} \,\left( {\text{g}} \right),$$

R2 $$3{\text{REPO}}_{4} \,\left( {\text{s}} \right) + 5{\text{Al}}\left( {{\text{OH}}} \right)_{3} \,\left( {\text{g}} \right) \to {\text{Al}}_{5} {\text{RE}}_{3} {\text{O}}_{12} \,\left( {\text{s}} \right) + 1.5{\text{P}}_{2} {\text{O}}_{5} \,\left( {\text{g}} \right) + 7.5{\text{H}}_{2} {\text{O}}\,\left( {\text{g}} \right),$$

R3 $$3{\text{REPO}}_{4} \,\left( {\text{s}} \right) + 2.5{\text{Al}}_{2} {\text{O}}_{3} \,\left( {\text{s}} \right) \to {\text{Al}}_{5} {\text{RE}}_{3} {\text{O}}_{12} \,\left( {\text{s}} \right) + 1.5{\text{P}}_{2} {\text{O}}_{5} \,\left( {\text{g}} \right).$$

As these chemical reactions would probably affects weight loss or gain during water vapor corrosion test of REPO_4_ bulk samples, we would firstly evaluate the potential effect on weight change of these reactions. According to Chemical Formula (R3), the ingest of alumina from the environment will lead to weight gain, whilst the formation of gaseous product P_2_O_5_ will cause weight loss. Hence, the weight change is dependent on these two factors. As the weight of 2.5 molar ingested Al_2_O_3_ is slightly higher than that of 1.5 molar gaseous P_2_O_5_, this reaction would result in a gentle increase of weight. According to Chemical Formula (R3), the reaction of 3 molar REPO_4_ will lead to a weight gain of 42 g. Table 6 shows weight gain percentage relative to the REPO_4_ mass according to a thorough reaction conforming to Formula (R3), i.e. all REPO_4_ has been consumed by alumina. As shown, given all REPO_4_ has been consumed to form Al_5_RE_3_O_12_, only a 5–10% weight gain would be generated. However, in reality, only a small amount of REPO_4_ reacts with Al_2_O_3_ from the alumina tube to form Al_5_RE_3_O_12_. Hence, a negligible weight gain, at least smaller than the error bar, would be generated due to the above byproduct formation. Therefore, the current obtained water vapor resistance data are still reliable.

**Table 6:** The weight gain and weight gain percentage relative to original REPO_4_ mass, assuming a thorough reaction according to Chemical Formula (R3), i.e. all REPO_4_ has been consumed by Al_2_O_3_.

	Weight of 3 molar REPO_4_ (g)	Weight gain (g)	Weigh gain percentage relative to REPO_4_ mass (%)
LuPO_4_	810	42	5.2
YbPO_4_	804	42	5.2
ErPO_4_	787	42	5.3
YPO_4_	551.7	42	7.6
ScPO_4_	420	42	10

**Figure 10:**
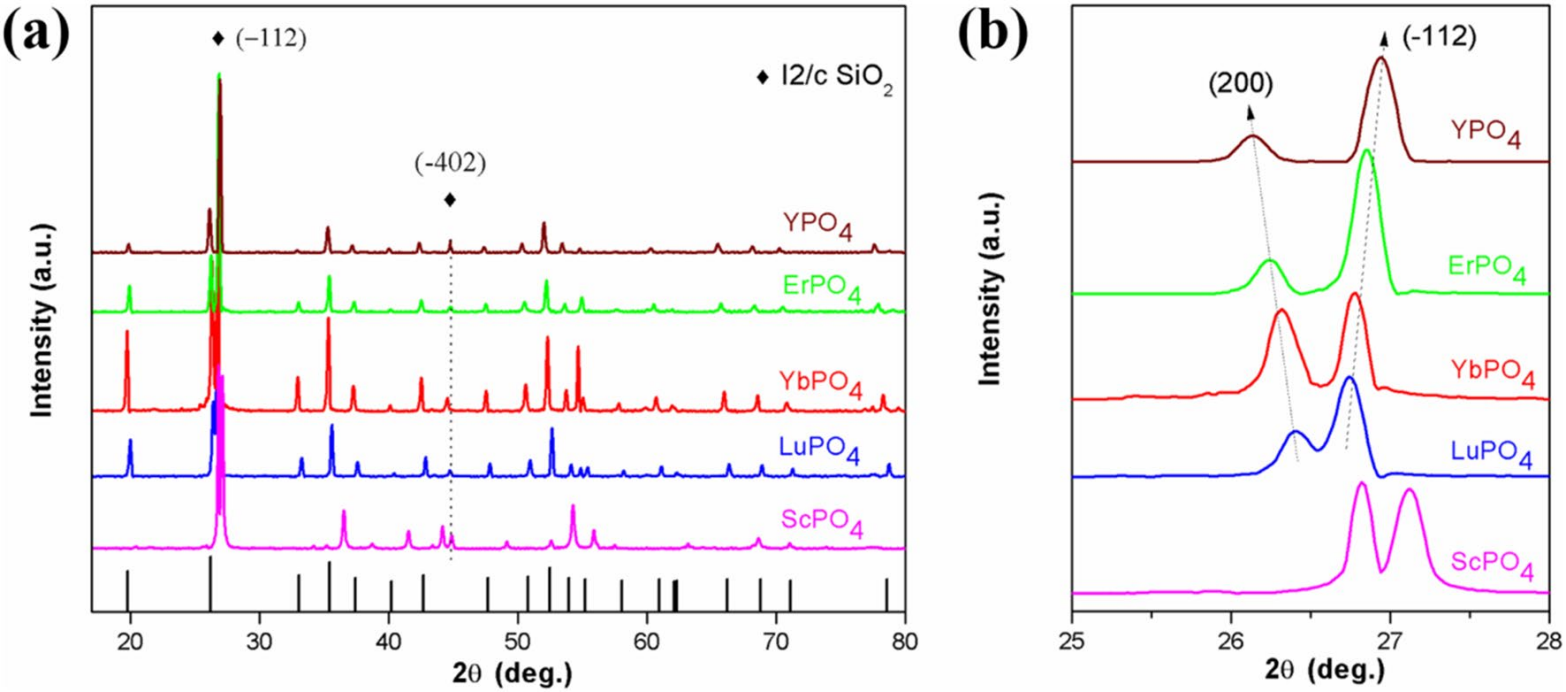
XRD patterns of REPO_4_ mixed with silicon after heat treatment at 1,350 °C for 20 h: (a) 17° ≤ 2θ ≤ 80°; (b) 25° ≤ 2θ ≤ 28°.

Finally, the chemical compatibility between REPO_4_ (RE = Lu, Yb, Er, Y and Sc) and Si bond layer is further investigated. After annealing of 30 wt.% silicon and REPO_4_ powders at 1,350 °C for 20 h, the powder mixtures are then subject to x-ray diffraction and their patterns are shown in Fig. 10. As shown, the characteristic peaks of Moganite-type SiO_2_, as indicated in Fig. 10a, have been present. In Fig. 10b, which is a high magnitude XRD pattern of 25° ≤ 2θ ≤ 28°, the XRD peaks of Xenotime-type REPO_4_ denoting (2 0 0) planes are systematically shifting towards lower 2θ angles with an increase of cationic radius of RE, which is consistent with Bragg’s law. By contrast, the peak positions of (-1 1 2) planes of Moganite-type SiO_2_ keep relatively unchanged. There are no impurity peaks except the above two phases, suggesting that the current investigated REPO_4_ is thermochemically compatible with the silicon bond coat.

## Conclusion

The mechanical and thermophysical properties of Xenotime-type REPO_4_ (RE = Lu, Yb, Er, Y and Sc) have been thoroughly investigated by first-principle calculations and experimental studies respectively with a potential environmental barrier coating application. The main conclusions are as follows. First, from calculations, large Poisson’s ratio and big B/G ratio are predicted for currently investigated rare earth phosphate compounds except ScPO_4_, suggesting that they have some sort of quasi-ductile behavior, which is perhaps beneficial to the durability and lifespan of their made coatings. Second, from the study of underlying parameters governing thermophysical properties of a ceramic, it suggests that, a low thermal expansion coefficient (which yields a good TEC matching of EBC with SiC-based CMC substrates) and a low thermal conductivity (which provides perhaps a good thermal insulation of EBC) are unfortunately exclusive, and thus not possible to achieve simultaneously. For EBC application, a good TEC match of topcoats and substrates is perhaps more important than thermal insulation properties, suggesting a weak lattice anharmonicity of a lattice might be beneficial. However, a weak lattice anharmonicity, i.e. strong lattice harmonicity might result in strong bonds, i.e. larger elastic modulus, which might be detrimental to the strain tolerance of its coatings. This suggests that a compromised value of TEC and thermal conductivities of a topcoat material is more favorable. In fact, the current studied REPO_4_ exhibits a very good TEC match with SiC-based CMCs, particularly for those heavier rare earth elements. Third, the current investigated REPO_4_ exhibits very good high-temperature water vapor corrosion resistance, excellent CMAS resistance as well as excellent chemical compatibility with silicon bond coats at elevated temperatures. By considering the above three aspects, it is proposed that Xenotime-type rare earth phosphates are a promising EBC topcoat material.
